# Supplementary material for: Dapagliflozin Restores Impaired Autophagy and Suppresses Inflammation in High Glucose-Treated HK-2 Cells
Source: Cells. 2021 Jun 10;10(6):1457. doi: 10.3390/cells10061457 (PMC8230404; doi:10.3390/cells10061457)
Supplement: Supplementary file 1 [file cells-10-01457-s001.zip › cells-1208122-supplementary.pdf]

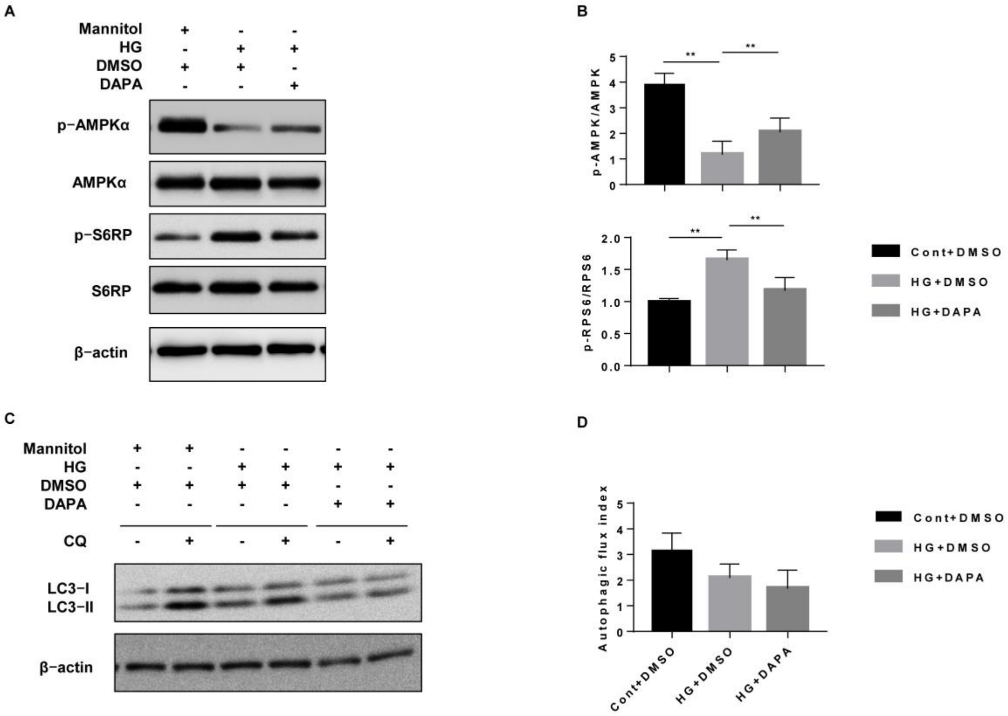

Figure S1. Short time treatment of dapagliflozin increased the activity of AMPK and but had no effect on autophagic flux. (**A**) HK-2 cells were treated with dapagliflozin (20 μM, for 24 h) under HG conditions, and p-AMPK, p-S6RP were assessed by western blotting. (**B**) Densitometric evaluation of (**A**) (n=4). (**C**) HK-2 cells were treated with Cont, HG (30 mM), HG (30 mM) with dapagliflozin (20 μM) for 24 h and chloroquine (50 μM) or vehicle for 1 h. Autophagic flux was determined with the LC3-II ratio in the presence and absence of chloroquine. (**D**) Densitometric evaluation of (C) (n=4). All data represent the means ± standard deviation (SD). *p<0.05 vs. the indicated group, **p<0.01 vs. the indicated group. Cont, control; HG, high glucose; DAPA, dapagliflozin; and CQ, chloroquine.

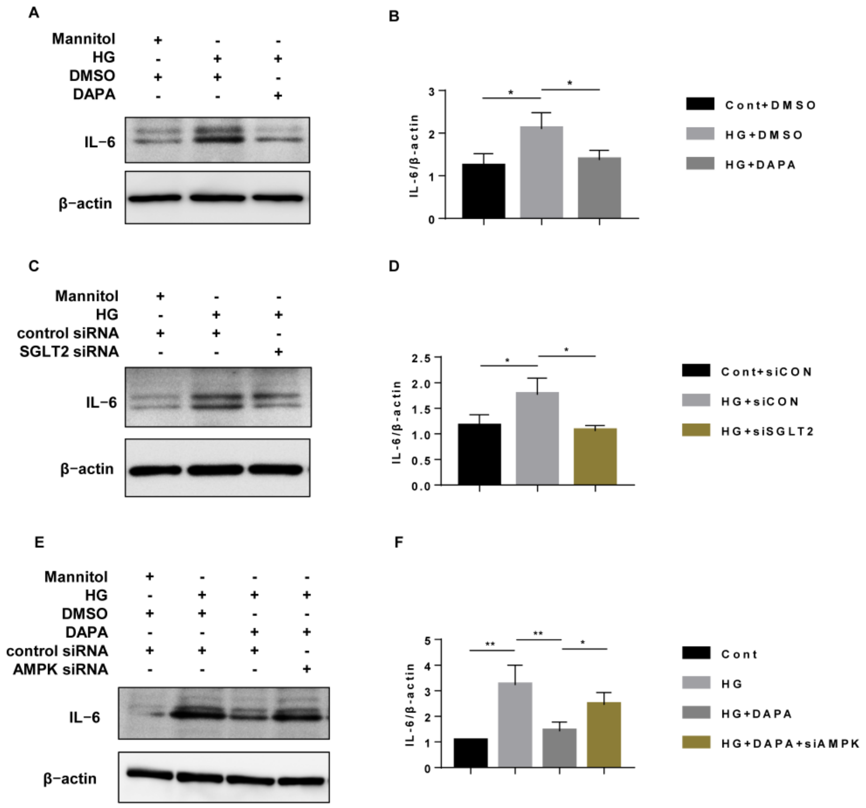

Figure S2. SGLT2 inhibition suppressed HG-induced IL-6 via activating AMPK. (**A**) HK-2 cells were incubated with Cont, HG (30 mM), or HG (30 mM) dapagliflozin (20 μM) for 48 h, and IL-6 was assessed by western blotting. (**B**) Densitometric evaluation of (A) (n=3). (**C**) HK-2 cells were transfected with SGLT2 siRNA (200 nM) or control siRNA for 6 h and then incubated with Cont or HG (30 mM) medium for 48 h. IL-6 was assessed by western blotting. (**D**) Densitometric evaluation of (A) (n=4). (**E**) HK-2 cells were transfected with AMPK siRNA (200 nM) or control siRNA for 6 h and then incubated with Cont, HG (30 mM) or HG (30 mM) with dapagliflozin (20 μM) for 48 h. The levels of IL-6 in cytoplasmic protein extracts were assessed by western blotting. (**F**) Densitometric evaluation of (E) (n=4). All data represent the means ± standard deviation (SD). *p<0.05 vs. the indicated group, **p<0.01 vs. the indicated group (n=3). Cont, control; HG, high glucose; siCON, control siRNA; siAMPK, AMPK siRNA.

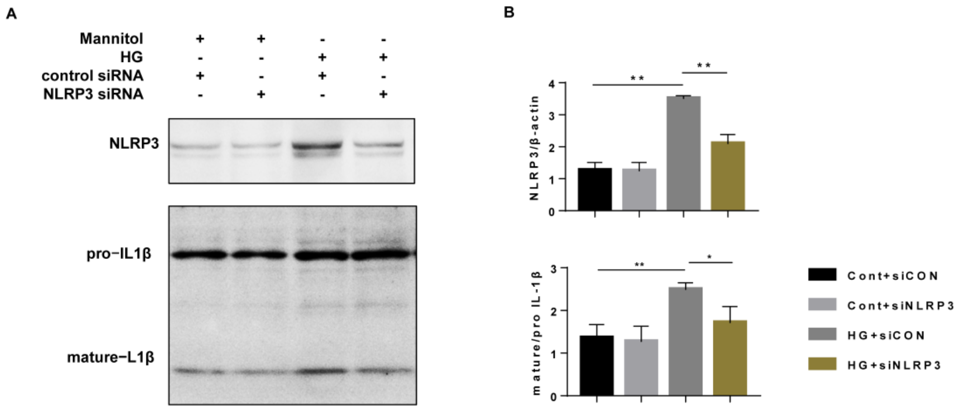

Figure S3. NLRP3 knockdown suppressed HG-induced activation of IL-1β. **(A)** HK-2 cells were transfected with NLRP3 siRNA (200 nM) or control siRNA for 6 h and then incubated with Cont or HG (30 mM) medium for 48 h. The levels of NLRP3 and IL-1β were assessed by western blotting. **(B)** Densitometric evaluation of (A) (n=3). All data represent the means ± standard deviation (SD). **p<0.01 vs. the indicated group. Cont, control; HG, high glucose; siCON, control siRNA; siNLRP3, NLRP3 siRNA.

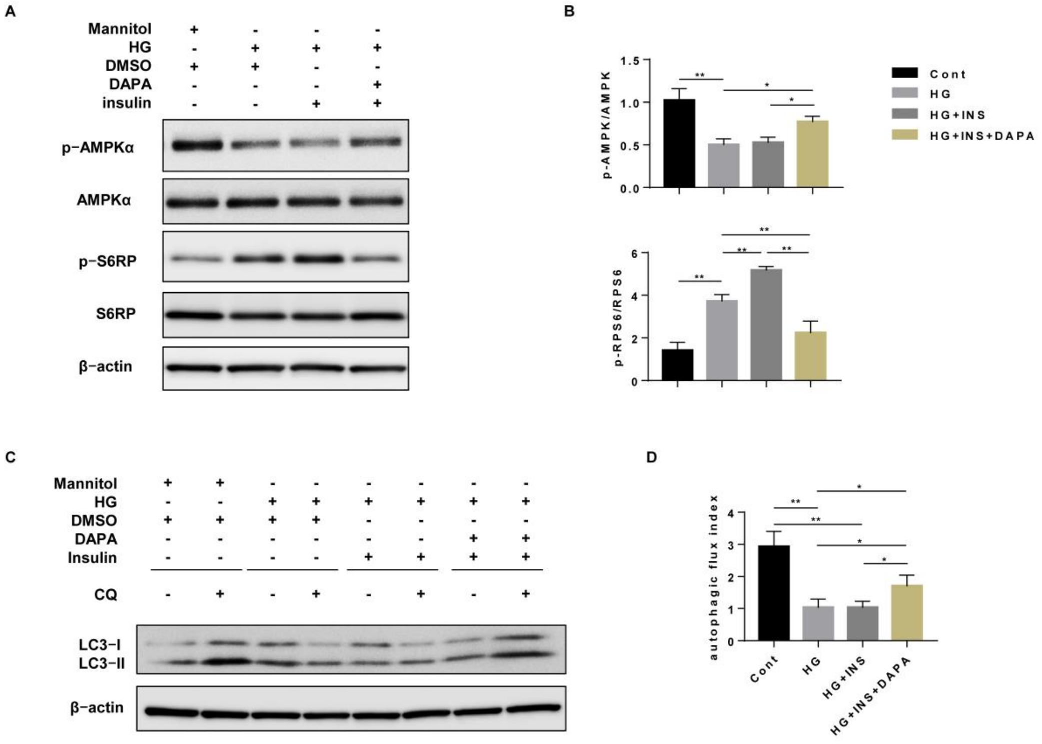

Figure S4. Dapagliflozin increased the activity of AMPK and autophagic flux in insulin-treated HK-2 cells. (**A**) HK-2 cells were treated with Cont, HG (30 mM), HG (30 mM) with insulin (100 nM), HG and insulin (100 nM) plus dapagliflozin (20 μM) for 48 h, and p-AMPK, p-S6RP were assessed by western blotting. (**B**) Densitometric evaluation of (A) (n=3). (**C**) HK-2 cells were treated with Cont, HG (30 mM), HG (30 mM) with insulin (100 nM), HG (30 mM) with insulin (100 nM) plus dapagliflozin (20 μM) for 48 h and chloroquine (50 μM) or vehicle for another 1 h. Autophagic flux was determined with the LC3-II ratio in the presence and absence of chloroquine. (**D**) Densitometric evaluation of (C) (n=5). All data represent the means ± standard deviation (SD). *p<0.05 vs. the indicated group, **p<0.01 vs. the indicated group. Cont, control; HG, high glucose; DAPA, dapagliflozin; INS, insulin; CQ, chloroquine.

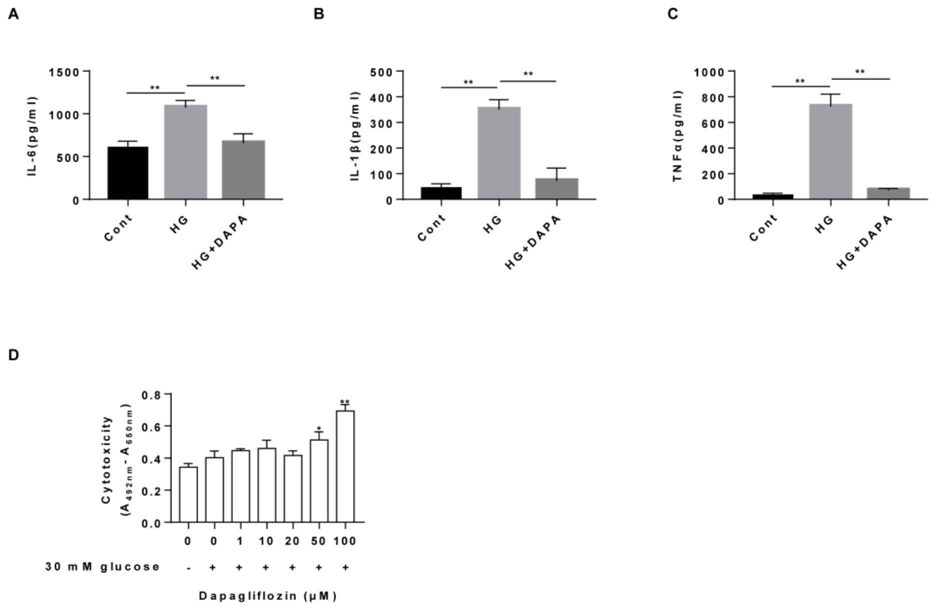

Figure S5. Dapagliflozin suppressed HG-induced pro-inflammatory cytokines in cultured medium. HK-2 cells were incubated with Cont, HG (30 mM), or HG (30 mM) dapagliflozin (20 μM) for 48 h, levels of pro-inflammatory cytokines in cultured medium including (**A**) IL-6, (**B**) IL-1β and (**C**) TNFα were mesured by ELISA (n=5). *p<0.05 vs. the indicated group, **p<0.01 vs. the indicated group. (**D**) Cytotoxicity of dapagliflozin on HG-treated HK-2 cells was assayed by LDH cytotoxicity detection kit after incubation with different concentrations of dapagliflozin (0-100 μM) for 48 h (n=3). *p<0.05 vs. 30 mM glucose group, **p<0.01 vs. 30 mM glucose group. Cont, control; HG, high glucose; DAPA, dapagliflozin.
